# Supplementary material for: Arabidopsis O‐GlcNAc transferase SEC activates histone methyltransferase ATX1 to regulate flowering
Source: EMBO J. 2018 Aug 27;37(19):e98115. doi: 10.15252/embj.201798115 (PMC6166131; doi:10.15252/embj.201798115)
Supplement: Supplementary file 1 — Appendix [file EMBJ-37-e98115-s001.pdf]

Appendix

Contents:

1. Appendix figure legends.
2. Appendix Fig S1
3. Appendix Fig S2
4. Appendix Fig S3
5. Appendix Table S1
6. Appendix Table S2
7. Appendix Table S3

Appendix Figure Legends

Appendix Figure S1. Loss of SEC function alters histone modification states on *FLC* chromatin.

A Scheme of the *FLC* locus. Exons are indicated with black boxes; promoter and introns are shown as lines. P1, P2 and P3 refer to genomic regions examined by ChIP.

B ChIP-qPCR assay of H3K4me2 levels of indicated regions at *FLC* chromatin of all three biological replicates corresponding to Fig 2B.

C ChIP-qPCR assay of H3K4me3 levels of indicated regions at *FLC* chromatin of all three biological replicates corresponding to Fig 2C.

D ChIP-qPCR assay of H3K36me3 levels of indicated regions at *FLC* chromatin of all three biological replicates corresponding to Fig 2D.

E ChIP-qPCR assay of H3K27me3 levels of indicated regions at *FLC* chromatin of all three biological replicates corresponding to Fig 2E.

Data information: For ChIP analysis, 12-d-old plants were collected and three independent experiments were conducted, and these three independent biological replicates correspond to Fig 2. The relative abundance was normalized to the input. Data are mean ± SD, n = 3. R1: replicate 1; R2, replicate 2; R3, replicate 3.

Appendix Figure S2. Induction of His-ATX1ΔN and His-SECΔN recombinant proteins.

A Diagram of ATX1, showing putative conserved domains in ATX1. The amino acid sequence shows ATX1-ΔN for recombinant expression, and the SET domain is in red.

B Diagram of conserved domains in SEC.

C Expression induction of His-ATX1ΔN and His-SECΔN. Recombinant proteins were induced by 1 mM isopropyl-β-D-thiogalactoside (IPTG) in *E. coli* strain BL21 (DE3). Lane M, molecular weight markers; lane 1, cell lysates before IPTG induction; lane 2, cell lysate induced with 1 mM IPTG at 15℃ for 16 h; lane 3, cell lysate induced with 1 mM IPTG at 37℃ for 4 h.

Appendix Figure S3. Mutation of S947A inhibits ATX1-activated *FLC* transcription.

Quantitative real-time PCR analysis of *ATX1* and *FLC* transcript levels in Col-0, *atx1-2*, *atx1-2 35S::ATX1-FLAG*, and *atx1-2 35S::ATX1m-FLAG* plants. The transcript levels were normalized to that of *TUBULIN*. Data shown are mean ± SD, n = 3. These three independent biological replicates correspond to Fig 6B. R1: replicate 1; R2, replicate 2; R3, replicate 3.

Appendix Fig S1

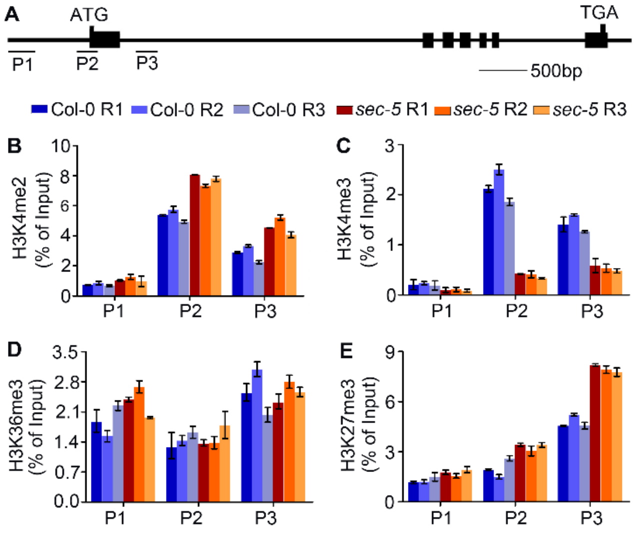

Appendix Fig S2

A

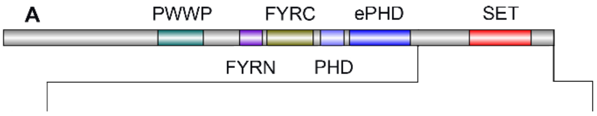

801 ATHKTSEYLP PPNPSGCART EPYNCFGRRG RKEPEALAAA
841 SSKRLFVENQ PYVIGGYSRL EFSTYKSIHG SKVSQMNTPS
881 NILSMAEKYR YMRETYRKRL AFGKSGIHGF GIFAKLPHRA
921 GDMMIEYTGE LVRPSIADKR EQLIYNSMVG AGTYMFRIDD
961 ERVIDATRTG SIAHLINHSC VPNCYSRVIT VNGDEHIIIF
1001 AKRHIPKWEE LTYDYRFFSI GERLSCSCGF PGCRGVVNDT
1041 EAEEQHAKIC VPRCDLIDWT AE

B

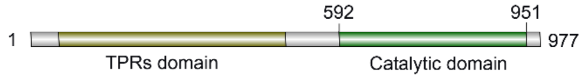

C

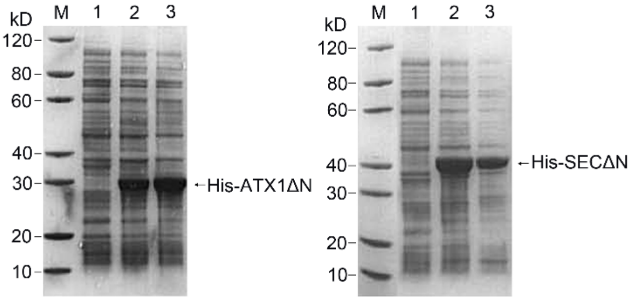

Appendix FigS3

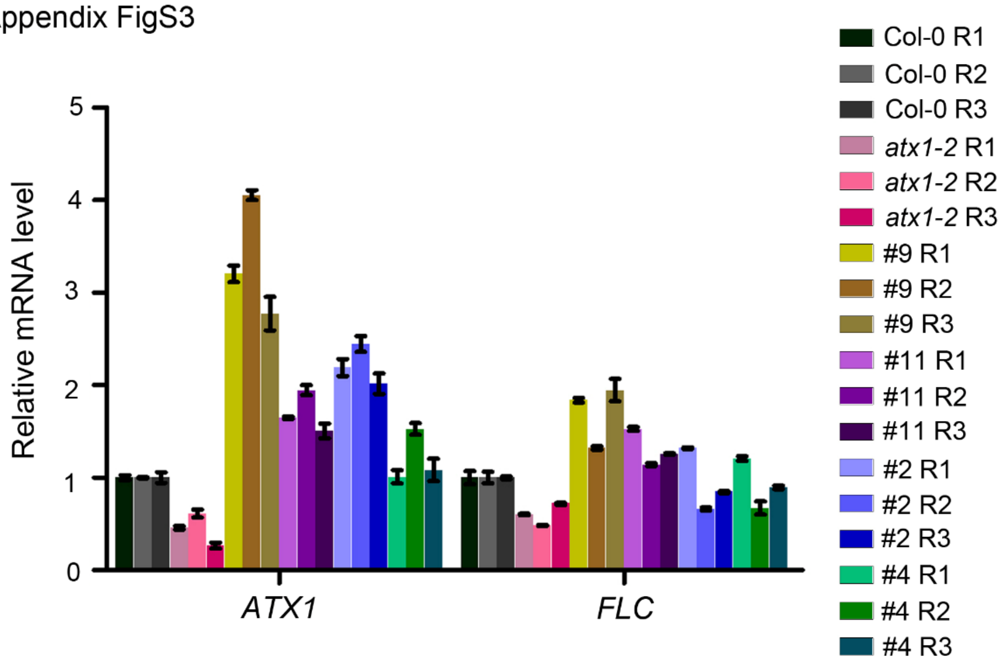

Appendix Table S1. The early flowering phenotype of the *sec-5* mutant under SD conditions.

	Genotype	Days to first flower opening	Rosette leaf no.	*n*
**SD+V0**	Col-0	85.3 ± 1.5	44.3 ± 2.9	60
	sec-5	72.3 ± 1.2*	33.5 ± 3.4**	60
**SD+V20**	Col-0	70.7 ± 2.5	31.1 ± 3.0	54
	sec-5	61.7 ± 1.2*	24.9 ± 3.3**	54

SD, short-day condition; V0, without vernalization; V20, vernalized for 20 days. (*), Significant differences at $P < 0.05$ between Col-0 and mutants by two tailed *t*-test. (**), Significant differences at $P < 0.01$ between Col-0 and mutants by two tailed *t*-test. *n*, total numbers of plants used in statistical analysis.

Appendix Table S2. The early flowering phenotypes of *sec-5* compared with *atx1-2*, *sec90 atx1-2*, and *flc-3* mutants.

**Genotype**	**Visible buds (%)**	**Bolting rates (%)**	**Days to first flower opening**	***n***
Col-0	22.1 ± 2.6	8.8 ± 2.4	32.3 ± 1.5	54
sec-5	62.8 ± 2.4**	45.9 ± 2.7**	29.3 ± 0.6*	54
atx1-2	62.2 ± 3.9**	49.6 ± 2.7**	29.0 ± 1.0*	54
sec-5 atx1-2	66.3 ± 3.6**	50.4 ± 2.8**	28.7 ± 1.2*	54
flc-3	68.4 ± 1.7**	51.8 ± 1.6**	28.0 ± 1.7*	54

Percentages of plants with visible buds were scored after seeds had germinated for 21 days, and bolting rates were calculated after seeds had germinated for 24 days. Experiments were repeated three times; the values are the means ± standard deviation (SD). (*), Significant differences at $P < 0.05$ between Col-0 and mutants by two tailed *t*-test. (**), Significant differences at $P < 0.01$ between Col-0 and mutants by two tailed *t*-test. *n*, total numbers of plants used in statistical analysis.

Appendix Table S3. Primers used in this study.

Primer name	Sequence (5'-3')	Note
LP-1	AAGGATCAGCTGTGAAGATGC	T-DNA for *sec-4*
RP-1	TTGTATGGGGAGAGCATCAAG	T-DNA for *sec-4*
LP-2	TCATGAATCAATCCTTGAGCC	T-DNA for *sec-5*
RP-2	TTTCGATGTCCCTTCTTTGTG	T-DNA for *sec-5*
LBb1.3	ATTTTGCCGATTTCGGAAC	T-DNA
SEC-qRT F	GATCAGTGTTTGGGATGCAC	qRT-PCR
SEC-qRT R	AGAAACGTCCAGGAAATGCT	qRT-PCR
SPY-qRT F	TTTCTGAAGCGATCAGGTTG	qRT-PCR
SPY-qRT R	CTGCTGCTGGCTTGTATGAT	qRT-PCR
ATX1-FLAG F SacI	gagctcATGGCGTGTTTTTCTAACGA	Construct
ATX1-FLAG R SalI	gtcgacCGGTCCAGTCTATTAGATCA	Construct
TUB-qRT F	ATCCGTGAAGAGTACCCAGAT	qRT-PCR
TUB-qRT R	AAGAACCATGTACTCATCAGC	qRT-PCR
FLC-qRT F	TAACCTGGTCAAGATCCTTGAT	qRT-PCR
FLC-qRT R	CAAGTTCAAGTAGCTCATAGTGTGA	qRT-PCR
SOC1-qRT F	GAGCAAGAAAGACTCAAGTGTTTAAGG	qRT-PCR
SOC1-qRT R	GAAGTGACTGAGAGAGAGAGAGTGAG	qRT-PCR
FLC-q P1F	AGGCGAGTGGTTCTTTGTTTTTAC	ChIP q-PCR
FLC-q P1R	CATGACGAGCGTCTTTGCTACTT	ChIP q-PCR
FLC-q P2F	CGACGAAGAAAAAGTAGATAGGCAC	ChIP q-PCR
FLC-q P2R	GTGACTTGTCGGCTACTTTTGTTCT	ChIP q-PCR
FLC-q P3F	CATGGATTTCATTATTTCCTTGG	ChIP q-PCR
FLC-q P3R	ATCCGAGAGATCCAATGAATTTTA	ChIP q-PCR
MAF1-qRT F	CAAACCTTTTATCTTCCTCTAGTGTGG	qRT-PCR
MAF1-qRT R	ACAAACTCTGATCTTGTCTCCGAA	qRT-PCR
MAF2-qRT F	TCTCTGGAGGAACAGCTCGAGACT	qRT-PCR
MAF2-qRT R	GAGCAGCGGAAGAGTCTCCCGTA	qRT-PCR
MAF3-qRT F	TCGGAATTATCTTCCACACAAGGAG	qRT-PCR
MAF3-qRT R	GCCAGAATCTGGTTCTCTTCTATCAGC	qRT-PCR
UBQ10-ChIPF	GGGCCTTGTATAATCCCTGATGAATAAGTG	qRT-PCR
UBQ10-ChIPR	AAAGAGATAACAGGAACGGAAACATAGT	qRT-PCR
SEC-FLAG F PstI	ctgcagATGATCTCGTCCAAAAACGG	Construct
SEC-FLAG-R SalI	gtcgacCATGTGGGAATTCTAGGTCG	Construct
ATX1-HA F SacI	gagctcATGGCGTGTTTTTCTAACG	Construct
ATX1-HA R SalI	gtcgacTTCTGCGGTCCAGTCTATT	Construct
AT3G04220 qF	GCTTGCTCTTTCCACAATGATGATA	qRT-PCR
AT3G04220 qR	AAGTTTTCCAGGTAAATAGTCCAGA	qRT-PCR
AT3G04230 qF	TCAATGTTTCGGAAGGAAGAAGACG	qRT-PCR
AT3G04230 qR	GGTAACTCTTCTGGAAACGAGCACG	qRT-PCR
AT3G04250 qF	TGTCGTAGCCACAAAGTCTTGAGGT	qRT-PCR
AT3G04250 qR	TTTATTATCCTCCTGCTTGGTTCGC	qRT-PCR

AT3G04260 qF	ATTGAAGAAGAAGAGGAGGAGGTCG	qRT-PCR
AT3G04260 qR	CAAGTTCTCCAAGGTCCAAACACAA	qRT-PCR
AD-SEC F ClaI	atcgatATGATCTCGTCCAAAAACGG	Construct
AD-SEC R SacI	gagctcTCTGTCATGTGGGAATTCTA	Construct
AD-SEC-N F NdeI	catatgATGATCTCGTCCAAAAACGG	Construct
AD-SEC-N R EcoRI	gaattcCCTCCTAATAATGCTTTCAA	Construct
BD-ATX1 F NcoI	ccattgATGGCGTGTTTTTCTAACGA	Construct
BD-ATX1 R SmaI	cccgggTTCTGCGGTCCAGTCTATTA	Construct
$_{SEC}$*pro*-F	AGACTTTCTTCTCCTCGTCA	*SEC* promoter
$_{SEC}$*pro*-R	CGCGTCTTAATGAAATTGAA	*SEC* promoter
ATX1-mF	CTCATCTACAATGCAATGGTGGGTG	Site mutation
ATX1-mR	CACCCACCATTGCATTGTAGATGAG	Site mutation
ATX1-qRT F	ATGGTTGTATTGGCAGCTACTTT	qRT-PCR
ATX1-qRT R	GATCCTCCTCCAGATACCTTGTT	qRT-PCR
