# Supplementary material for: Arabidopsis O‐GlcNAc transferase SEC activates histone methyltransferase ATX1 to regulate flowering
Source: EMBO J. 2018 Aug 27;37(19):e98115. doi: 10.15252/embj.201798115 (PMC6166131; doi:10.15252/embj.201798115)
Supplement: Supplementary file 2 — Expanded View Figures PDF [file EMBJ-37-e98115-s002.pdf]

Expanded View Figures

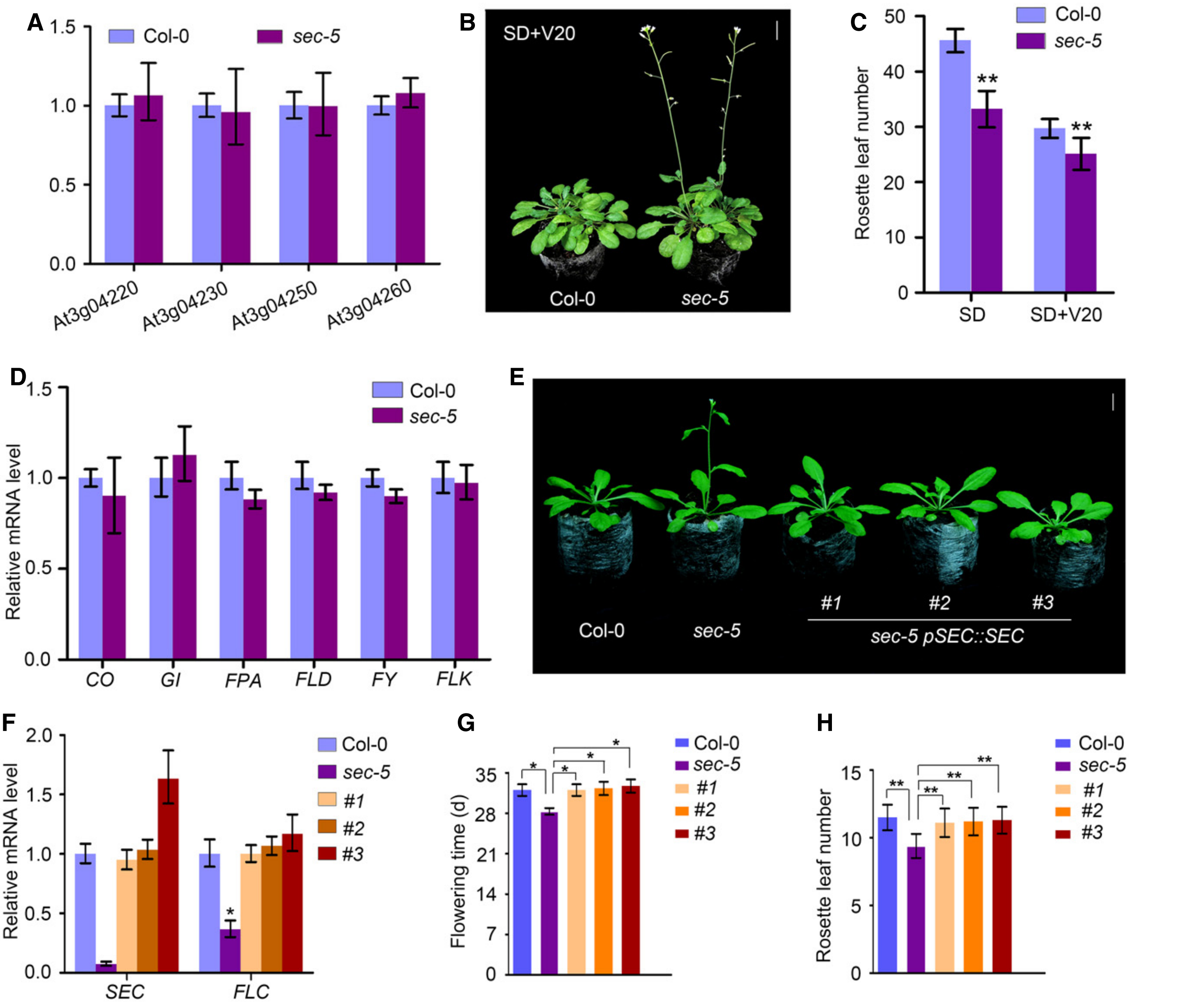

Figure EV1. Characterization and flowering phenotype of *sec-5* under SD conditions with vernalization, and flowering phenotype rescue of *sec-5*.

A qRT–PCR analysis of the expression of genes upstream and downstream of *SEC* in the *sec-5* mutant. The expression level was normalized to that of *TUBULIN*. Experiments were repeated three times, and one representative result is shown here. Data are mean ± s.d., $n = 3$.

B Flowering phenotype of Col-0 and *sec-5* plants with vernalization treatment under SD conditions. Plants were vernalized for 20 days before being moved to SD conditions. Scale bar: 1 cm.

C Rosette leaf number analysis of Col-0 and *sec-5* plants under SD with or without vernalization treatment. A total number of 62 plants were scored for rosette leaf number analysis of each line. Data are mean ± s.d., statistical significance (two-tailed *t*-test) with $^{**}P < 0.01$.

D qRT–PCR analysis of *CO*, *GI*, *FPA*, *FLD*, *FY*, and *FLK* mRNA expression levels in Col-0 and *sec-5* plants under LD conditions. The expression level was normalized to that of *TUBULIN*. Data are mean ± s.d., $n = 3$. Experiments were repeated three times, and one representative result is shown here.

E The early flowering phenotype of the *sec-5* mutant was rescued by *pSEC::SEC* under LD conditions. Three independent transgenic lines are shown. Scale bar: 1 cm.

F qRT–PCR analysis of *SEC* and *FLC* transcription levels in Col-0, *sec-5* and *pSEC::SEC* transgenic lines in a *sec-5* background. The expression level was normalized to that of *TUBULIN*. Data are mean ± s.d. of three independent biological replicates, $n = 3$. Statistical significance (two-tailed *t*-test) with $^{*}P < 0.05$.

G Flowering-time analysis of plant lines in (E). Error bars indicate s.d. of three biological replicates. Statistical significance (two-tailed *t*-test) with $^{*}P < 0.05$.

H Rosette leaf number analysis of plant lines in (E). Three biological replicates were conducted, and a total number of 52 plants were scored for rosette leaf number analysis. Error bars indicate s.d. Statistical significance (two-tailed *t*-test) with $^{**}P < 0.01$.

© 2018 The Authors

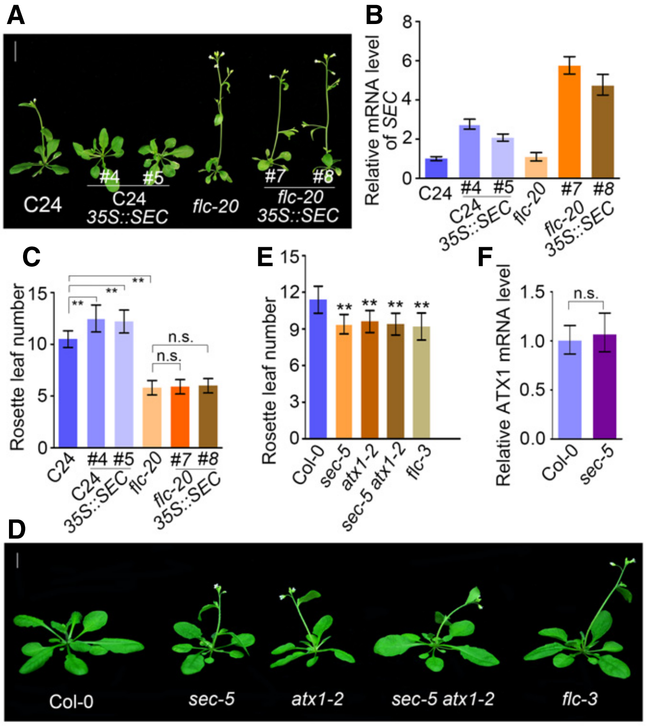

Figure EV2. Genetic interaction analysis between *SEC* and *FLC*, and flowering phenotype comparison between Col-0, *sec-5*, *atx1-2*, *sec-5 atx1-2*, and *flc-3* plants.

A Flowering phenotype of C24, C24 *35S::SEC*, *flc-20*, and *flc-20 35S::SEC* plants.

B Relative *SEC* mRNA expression levels of lines in (A). The expression level was normalized to that of *TUBULIN*. Data are mean ± s.d., $n = 3$. Experiments were repeated three times, and one representative result is shown here.

C Rosette leaf number analysis of plants in (A). The values are mean ± s.d.; a total of 40 plants were scored for each genotype. Statistical significance (two-tailed *t*-test) with $^{**}P < 0.01$.

D Flowering phenotype of Col-0, sec-5, *atx1-2*, *sec-5*, *sec-5 atx1-2*, and *flc-3* plants. Scale bar: 1 cm.

E Rosette leaf numbers of plant lines in (D). The values are mean ± s.d.; a total number of 62 plants were scored for rosette leaf number analysis. Statistical significance (two-tailed *t*-test) with $^{**}P < 0.01$.

F Expression level of *ATX1* mRNA in Col-0 and *sec-5* plants. Twelve-day-old plants were used for total RNA extraction. Three independent biological replicates were done, and one representative result is shown here. Data are mean ± s.d., $n = 3$. The data were normalized to that of *UBIQUITIN*. *ATX1* mRNA levels were analyzed for significant differences by two-tailed *t*-test. n.s., not significant.

© 2018 The Authors

A

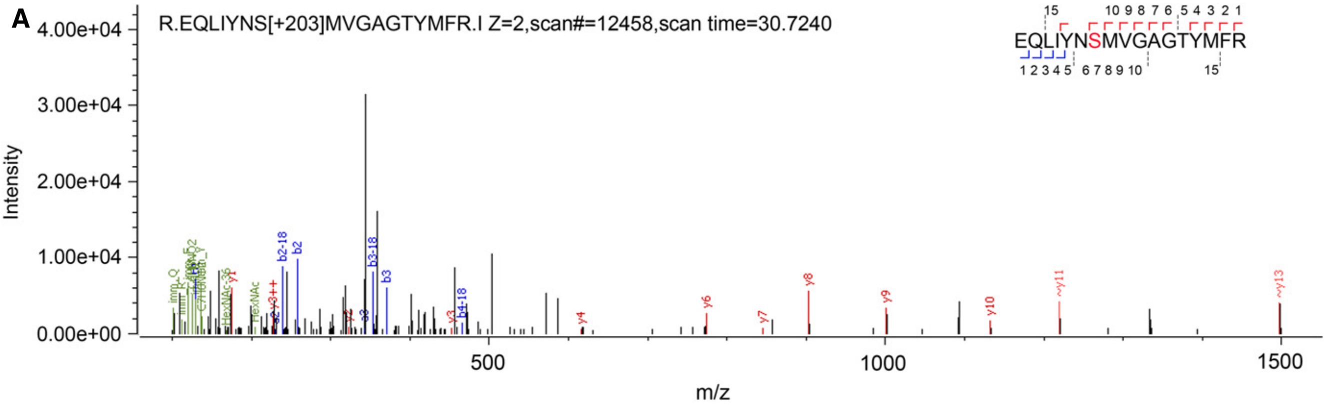

B

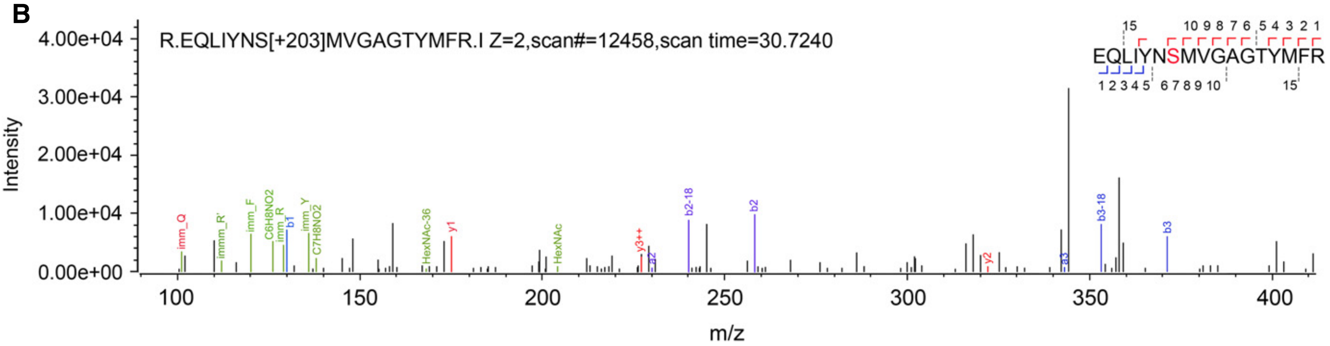

Figure EV3. Identification of potential *O*-GlcNAcylation site(s) in ATX1 SET domain.

A The mass spectrometry (MS) analysis showed that the serine and/or threonine residue(s) in the EQLIYNSMVGAGTYMFR peptide are (is) modified with *O*-GlcNAc.

B Partial enlarged detail of (A) from 100 to 400 of m/z.

A

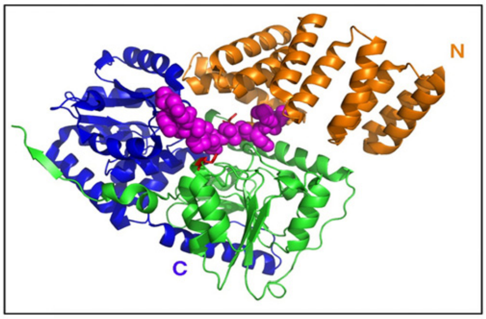

B

HsOGT 490 VSIVADQLEKNRLPSVHPHHSMLYPLSHGFRK
SEC 522 ESIIRRQINMSVLPSVQPFHAIAYPIDPILALEIS

HsOGT 555 RLRVGYVSSDFGNHPTSHLMQSIPGMHNPDK
SEC 591 RLRIGYVSSDFGNHPLSHLMGSVFGMHNREN

HsOGT 830 TTRSQYGLPEDAIVYCNFNQLYKIDPSTLQMW
SEC 757 PKRSDYGLPEDKFIFACFNQLYKMDPEIVNTW

Figure EV4. SEC structure and functionally conserved amino acids.

A SEC structure model. SEC N-terminal TPR domain (partial) is colored in gold (residues 50–538), residues 510–754 in green, and the C-terminal domain (residues from 755 to end) in blue. The magenta sphere represents the peptide binding to human OGT.

B Analysis of conserved amino acids for substrate binding of HsOGT and SEC. Conserved residues are shown in red.

© 2018 The Authors

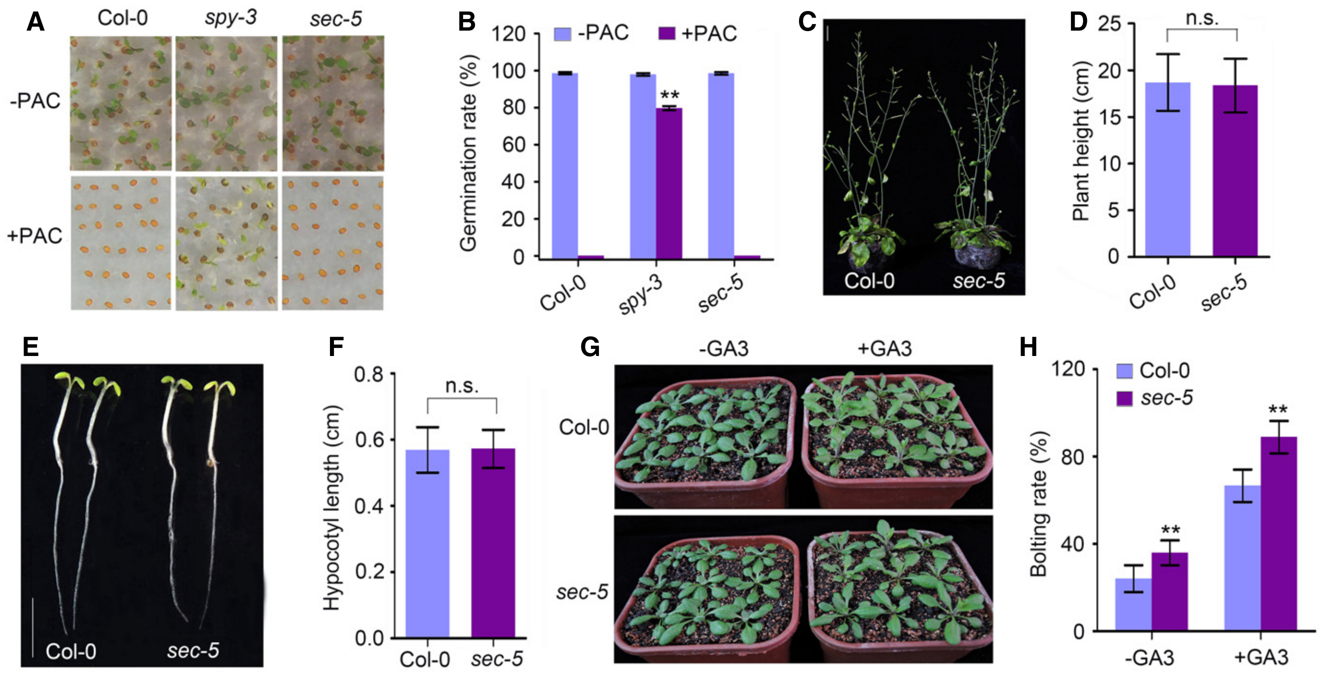

Figure EV5. Response to paclobutrazol and GA3 treatment of Col-0 and *sec-5* plants.

A The *sec-5* mutant shows a wild-type response to paclobutrazol (PAC).

B Germination rate analysis of Col-0, *spy-3*, and *sec-5* seeds under PAC treatment. Experiments were repeated three times. Errors bars are s.d. Two asterisks (**) indicate significant differences between Col-0 and *spy-3* plants ($P < 0.01$); *P*-value was determined by two-tailed *t*-test.

C, D Comparison of plant height between Col-0 and *sec-5* plants. Experiments were repeated three times. Error bars are s.d. More than 20 plants were analyzed. n.s., not significant by two-tailed *t*-test. Scale bar: 1 cm.

E Comparison of hypocotyl length of Col-0 and *sec-5* plants. Plants were grown under SD conditions. Scale bar: 0.5 cm.

F Statistical analysis of hypocotyl length of plants in (E). Hypocotyl length was measured after plants were grown under SD conditions for 5 days, and more than 20 plants were calculated for each line. Experiments were repeated three times. Error bars are s.d., n.s., not significant by two-tailed *t*-test.

G Comparison of flowering phenotype of Col-0 and *sec-5* plants with or without GA3 treatment.

H Calculation of bolting rate of Col-0 and *sec-5* plants at 20 days after plant germination. Experiments were repeated three times, and more than 20 plants were calculated for each line. Error bars are s.d. Two asterisks (**) indicate a significant difference between Col-0 and *sec-5* plants ($P < 0.01$); *P*-value was determined by two-tailed *t*-test.

 © 2018 The Authors
