# Supplementary figures and images for: Arabidopsis O‐GlcNAc transferase SEC activates histone methyltransferase ATX1 to regulate flowering
Source: EMBO J. 2018 Aug 27;37(19):e98115. doi: 10.15252/embj.201798115 (PMC6166131; doi:10.15252/embj.201798115)

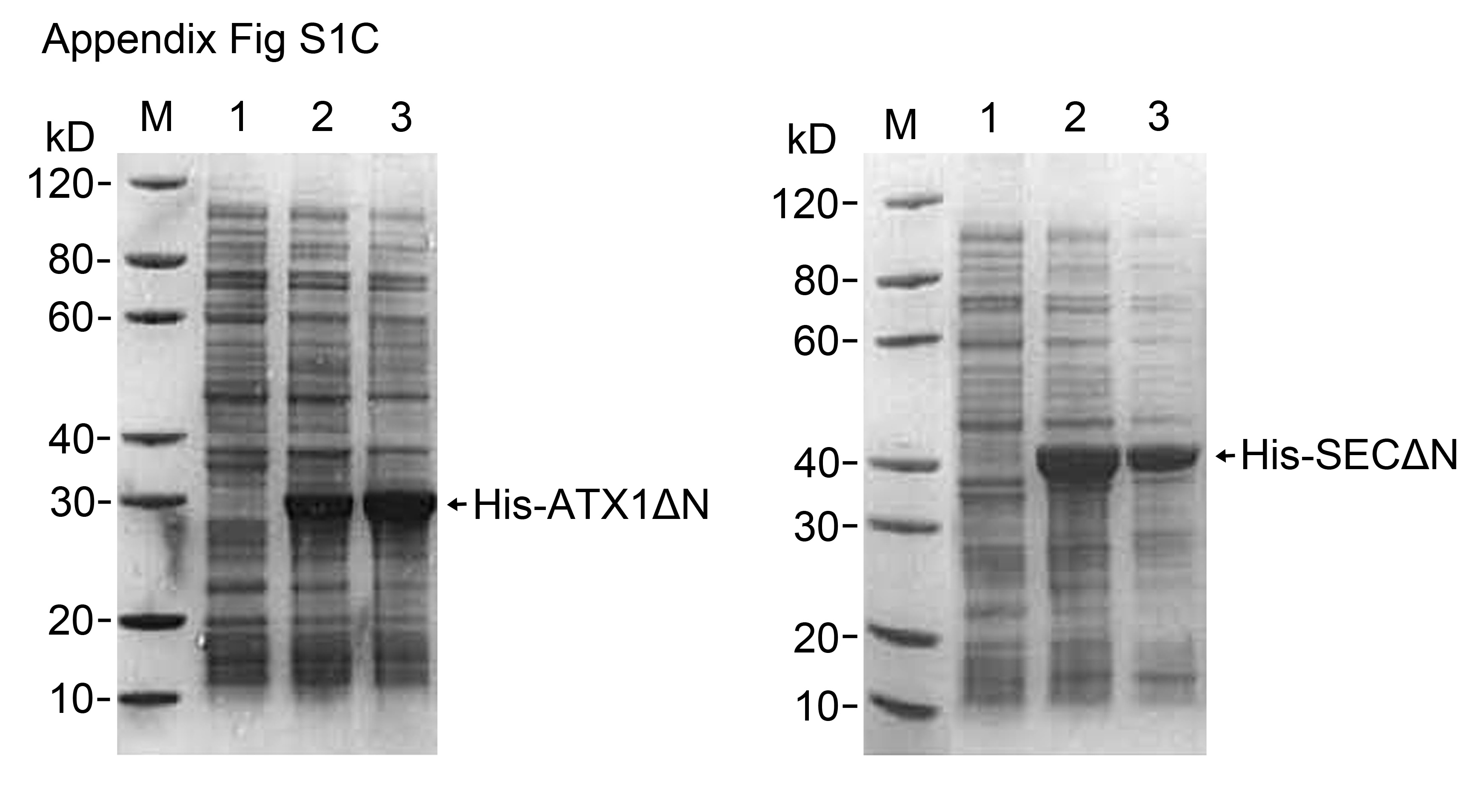

Supplement: Supplementary file 3 — Source Data for Expanded View [file EMBJ-37-e98115-s007.zip › embj201798115-sup-0003-SDataEV.jpg]

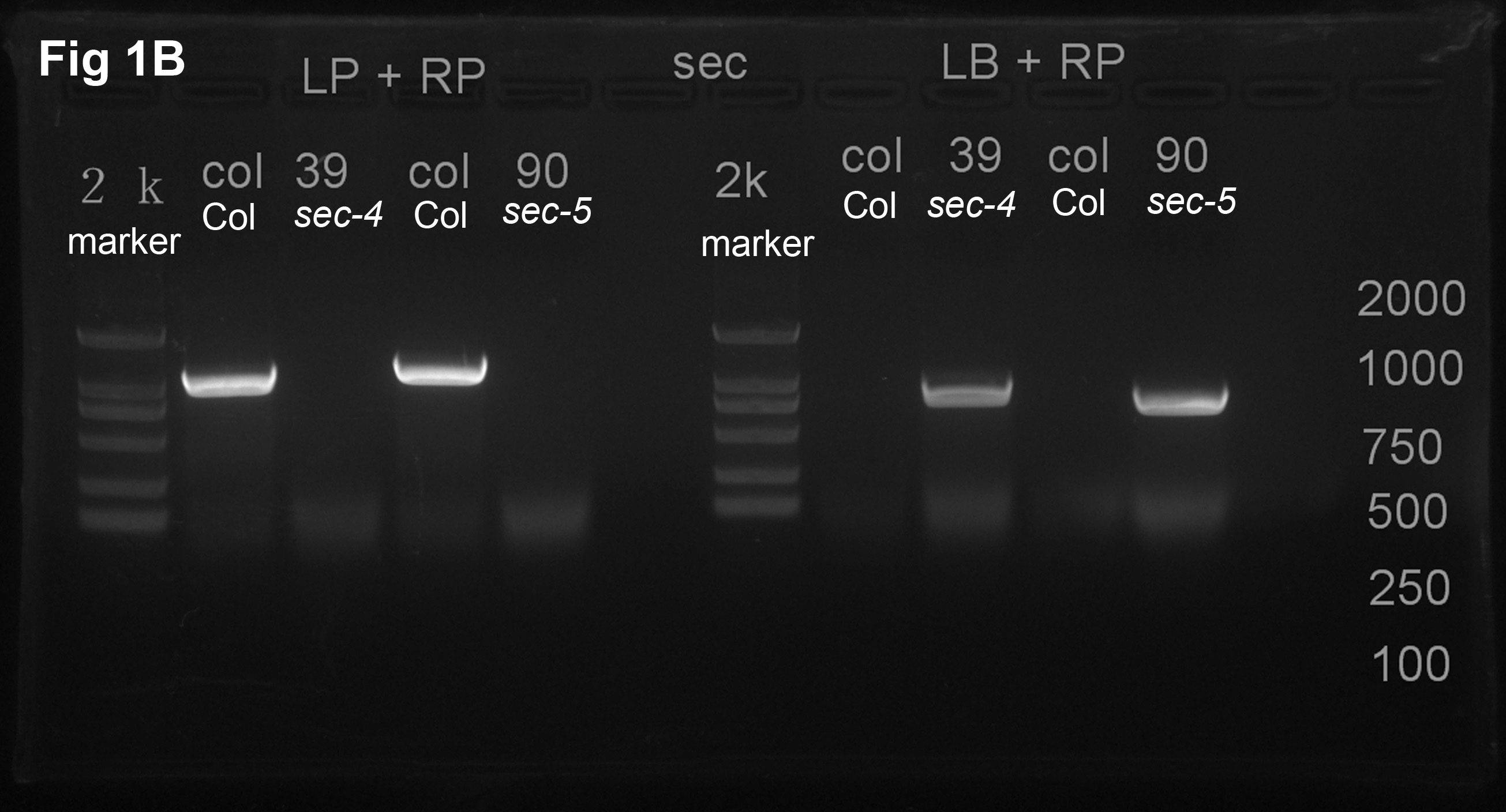

Supplement: Supplementary file 5 — Source Data for Figure 1 [file EMBJ-37-e98115-s003.tif]

**Fig 3B**

**Top**

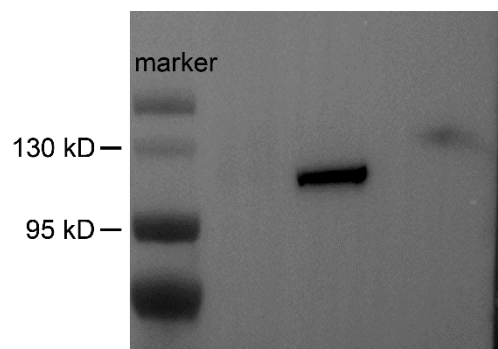

**Middle**

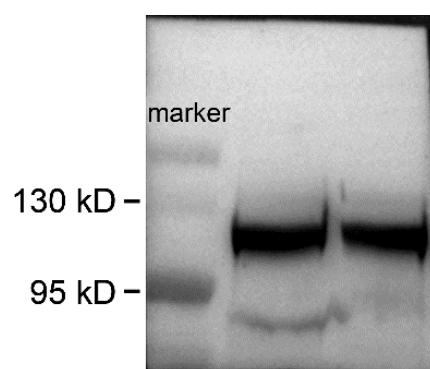

**Bottom**

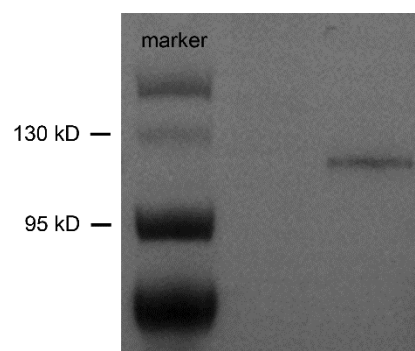

Supplement: Supplementary file 6 — Source Data for Figure 3 [file EMBJ-37-e98115-s004.pdf]

Fig 4A

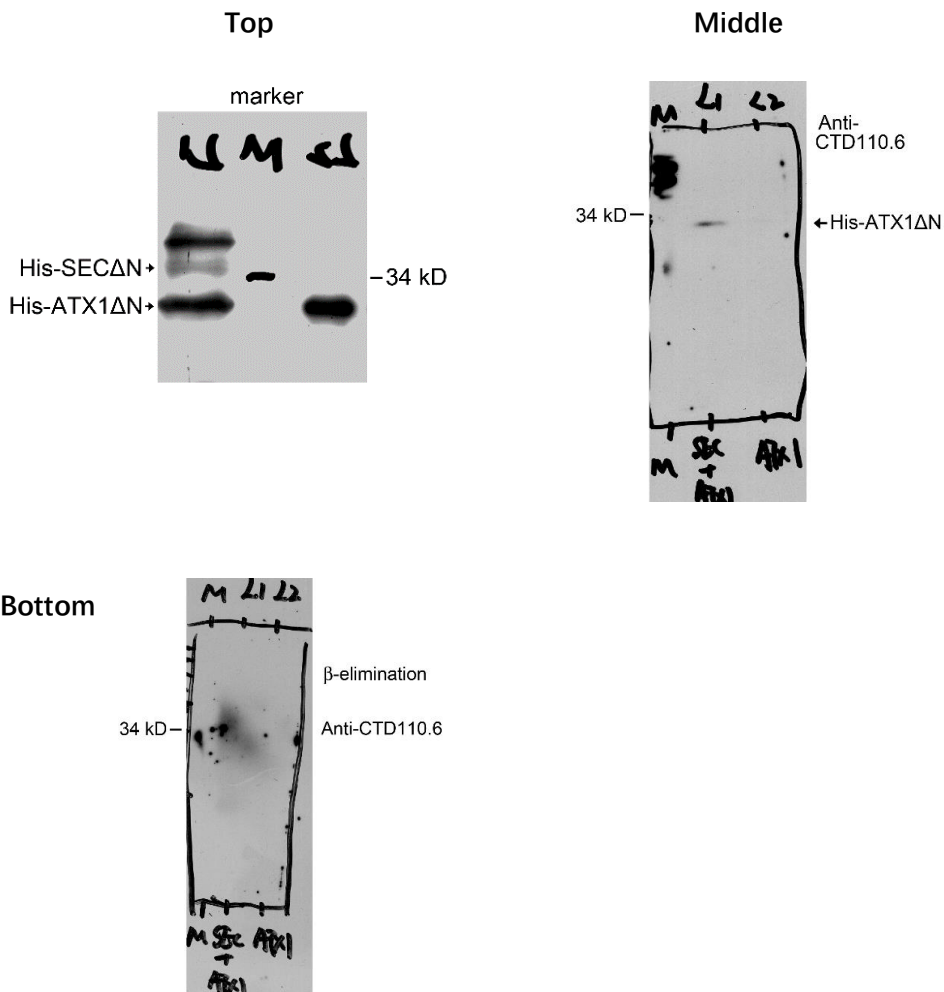

Fig 4B

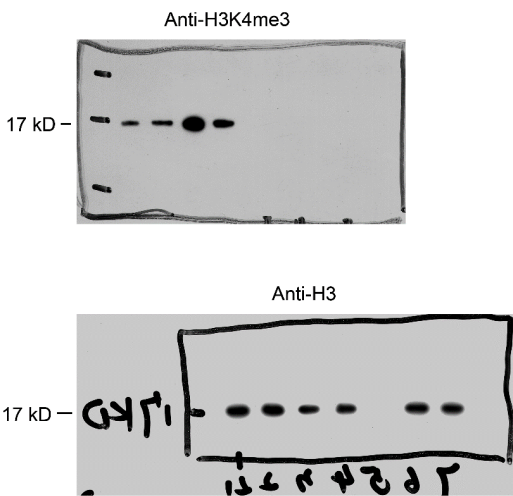

**Fig 4C**

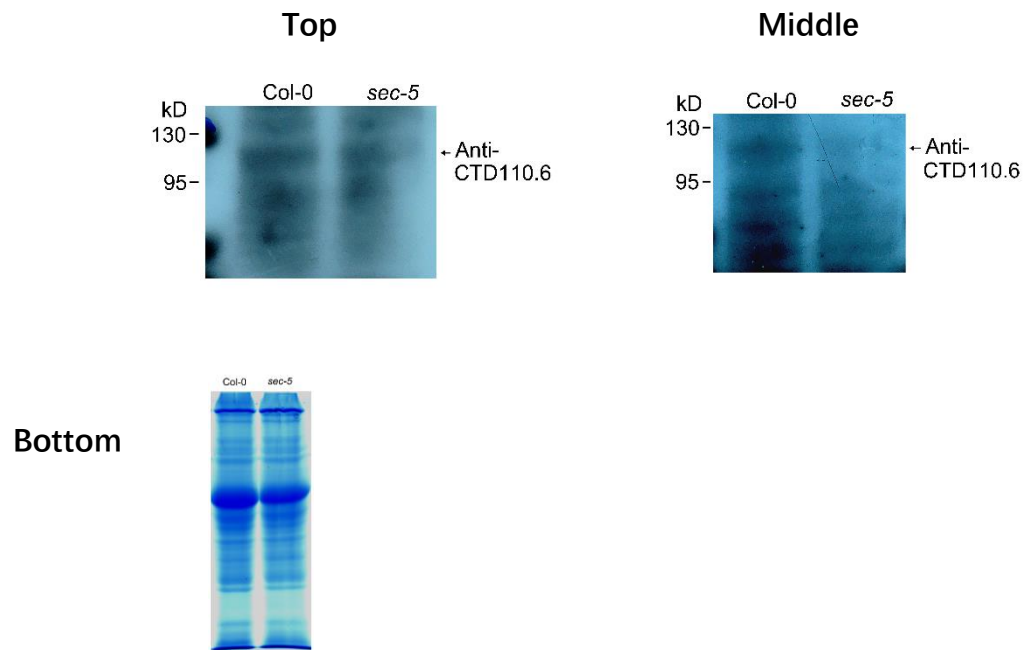

**Fig 4D**

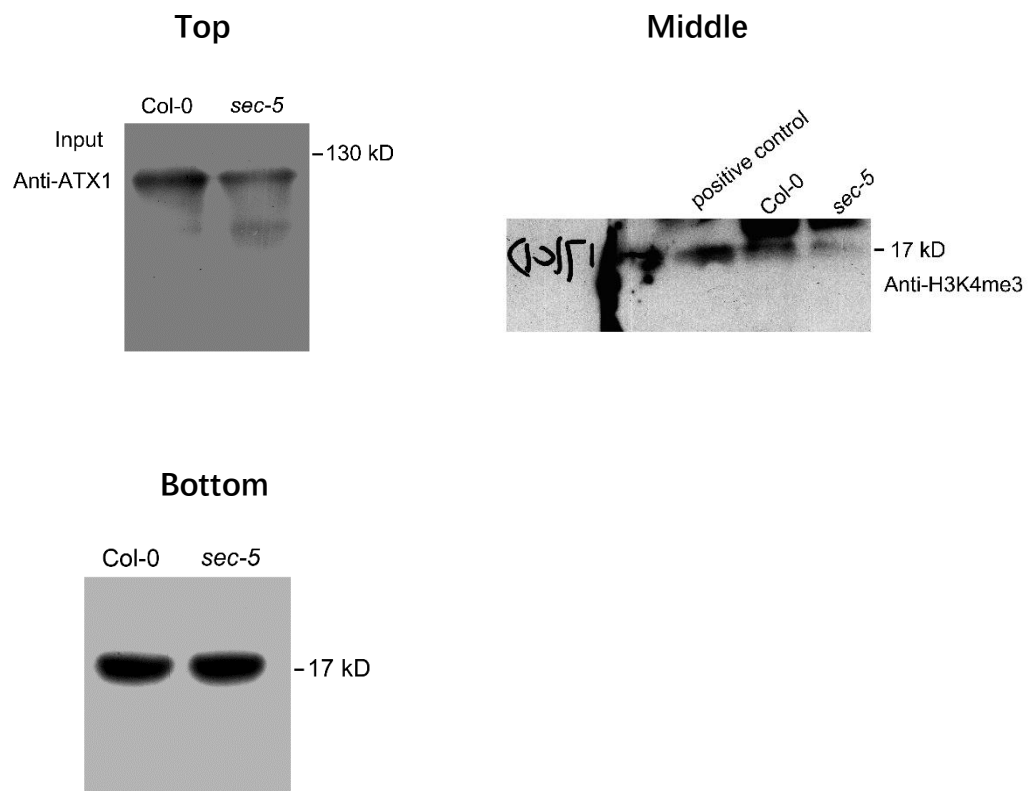

Fig 4E

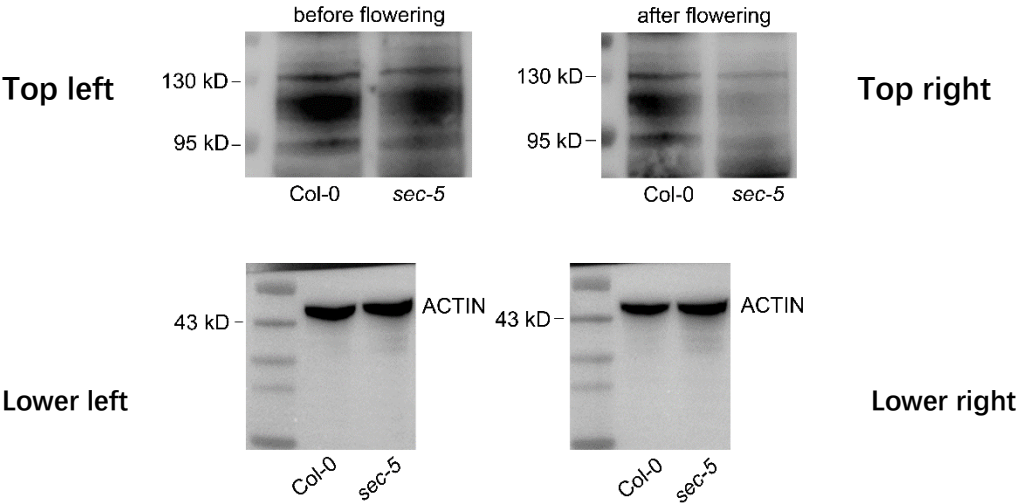

Supplement: Supplementary file 7 — Source Data for Figure 4 [file EMBJ-37-e98115-s005.pdf]

Fig 5A

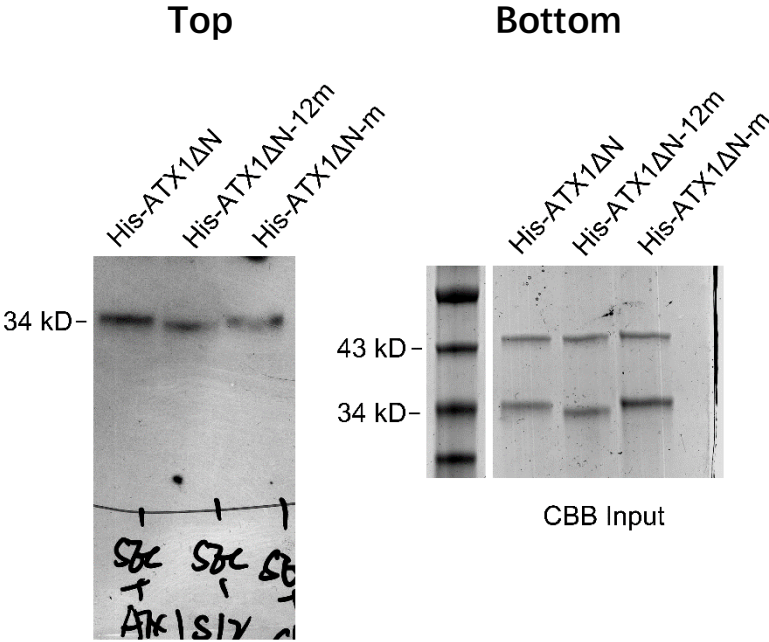

Fig 5B

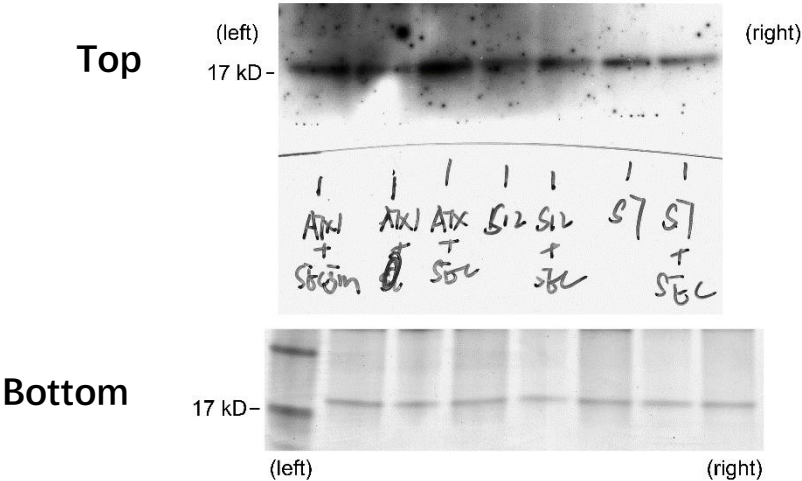

Supplement: Supplementary file 8 — Source Data for Figure 5 [file EMBJ-37-e98115-s006.pdf]
